# Analysis of epidemiological characteristics and vaccine effectiveness of pertussis in Linping District, China

**DOI:** 10.3389/fpubh.2026.1756836

**Published:** 2026-03-12

**Authors:** Tiane Liu, Qinghua Chen, Chunmei Ye, Jie Shen, Chang Zhu, Pai Yang, Rongrong Han, Chuandi Zhang

**Affiliations:** 1 Department of Expanded Program on Immunization, Hangzhou Linping District Center for Disease Control and Prevention (Hangzhou Linping District Health Supervision Institute), Hangzhou, Zhejiang, China; 2 Department of Public Health, Hangzhou Linping District Hospital of Integrated Traditional Chinese and Western Medicine, Hangzhou, Zhejiang, China

**Keywords:** case-control study, epidemiological characteristics, pertussis, pertussis vaccine, vaccine effectiveness

## Abstract

**Background:**

The increases in pertussis outbreaks among both vaccinated and unvaccinated individuals raises questions about the effectiveness of the pertussis vaccine (PV), highlighting the need for research on the impact of PV immunization on the incidence of pertussis in children.

**Methods:**

Descriptive epidemiological methods were used to analyze pertussis cases reported in Linping District from 2022 to 2023. A nested case-control study was conducted (1:4 matching). Conditional logistic regression was employed to calculate odds ratios (ORs) and vaccine effectiveness (VE) of PV.

**Results:**

A total of 149 cases of pertussis cases were reported in Linping District from 2022 to 2023, with an overall incidence rate of 6.20 per 100,000 population. The epidemic showed a unimodal pattern, peaking from April to June 2022. The majority of cases (99.33%) were children under 18 years old, with the highest proportion in the 0-year-old group (23.49%). Of the cases, 85.23% had a history of PV vaccination, and 73.15% had completed four doses. The case-control study revealed that the VE for any PV history versus no history was 92.86% (*95% CI*: 65.62% ~ 98.52%). The VE for 1 ~ 3 doses of basic immunization was 93.84% (*95% CI*: 68.10% ~ 98.81%). The VE for immunization within less than 1 year was 94.61% (*95% CI*: 71.08% ~ 99.00%). The VE for DTwP, DTaP, and DTaP-IPV/Hib vaccination was 92.68% (*95% CI*: 60.30% ~ 98.65%), 91.69% (*95% CI*: 59.13% ~ 98.31%), and 94.14% (*95% CI*: 70.45% ~ 98.84%), respectively.

**Conclusion:**

Pertussis primarily affected children in Linping District, showing a distinct spring peak. Delayed or missed vaccination was a key risk factor for infant cases. Under high vaccination coverage, four doses of DTaP vaccines provide good protection against pertussis at least through preschool age.

## 1 Introduction

Pertussis (whooping cough), caused by *Bordetella pertussis* (Bp), is a highly contagious acute respiratory infection that primarily infects the ciliated epithelial cells of the human respiratory tract through droplet transmission. It is characterized by paroxysmal spasmodic coughing accompanied by inspiratory whooping sounds and increased peripheral blood lymphocytes. The clinical manifestations of pertussis are diverse, often atypical, and can even be asymptomatic, facilitating its transmission and spread within households or other settings. As a result, individuals with pertussis, particularly those who are asymptomatic, can become the primary source of infection for unvaccinated or incompletely vaccinated infants and young children (1, 2). Commonly referred to as the “100-day cough” in folklore, pertussis can have a prolonged course, with symptoms lasting 2–3 months or even longer if left untreated. Delayed or inadequate treatment can lead to severe complications such as pneumonia and pertussis encephalopathy, which may pose life-threatening risks. This imposes a significant burden on infants and children (3).

Vaccination is one of the most cost-effective and impactful interventions for preventing infectious diseases in humans. Since the global implementation of expanded immunization programs in 1974, the incidence of pertussis has decreased by over 90% compared to levels observed in 1980, largely due to the steady increase in the coverage rate of the diphtheria, tetanus and whole-cell pertussis combined vaccine (DTwP) (4, 5). Due to the high rate of adverse reactions associated with DTwP vaccine, many countries have transitioned to using diphtheria, tetanus and acellular pertussis combined vaccine (DTaP) (6). In China, DTaP was included in the expanded National Immunization Program in 2007 and completely replaced DTwP in 2013. Over the past decade, the coverage rate for the 3-doses DTaP series in China has consistently exceeded 99% (7). A 40-year cohort study demonstrated that, due to the widespread use of pertussis vaccine (PV), pertussis morbidity and mortality decreased by 92.57 and 97.43%, respectively, from 1978 to 2017 (8).

Currently, three different acellular vaccines are used in Linping District to prevent pertussis: the DTaP, the diphtheria, tetanus and acellular pertussis—*Haemophilus influenzae* type b combined vaccine (DTaP-Hib), and the adsorbed diphtheria, tetanus and acellular pertussis—inactivated poliomyelitis—*Haemophilus influenzae* type b combined vaccine (DTaP-IPV/Hib) (9). According to the Vaccine Administration Law of the People’s Republic of China issued by the State Council, vaccines in China are categorized into two groups: National Immunization Program (NIP) vaccines and non-NIP vaccines. NIP vaccines are provided free of charge, while non-NIP vaccines require out-of-pocket payment (10). Non-NIP vaccines serve as effective supplements to NIP vaccines, offering more extensive and comprehensive protection for public health. These include DTaP-Hib and DTaP-IPV/Hib, which have replaced DTaP in China and other countries (11, 12). These vaccines provide protection against multiple vaccine-preventable diseases (VPDs), including: pertussis, diphtheria, tetanus, poliomyelitis (polio), and invasive diseases caused by Hib. They have garnered attention due to their ability to reduce the number of required doses, lower costs, and decrease manpower and resource requirements. Additionally, this approach can improve compliance and increase immunization coverage (13). In China, both DTaP and DTaP-Hib vaccines are prepared through the co-purification of two main components, pertussis toxin (PT) and filamentous hemagglutinin (FHA). In contrast, the component-purified DTaP-IPV/Hib vaccine is individually purified to contain two antigens: PT and FHA (14). The situation in Linping District aligns with the national practice. While few countries worldwide employ co-purified DTaP, it is of critically important to evaluate the vaccine effectiveness (VE) of co-purified DTaP. Research conducted in China has revealed that VE is positively correlated with the number of administered co-purified DTaP doses. Specifically, pertussis-related illness and hospitalization rates increased significantly, ranging from 24 to 26% after one dose to 86–87% after four doses (15).

In China, although PV coverage has continued to improve, with vaccination rates as high as 99% in children under 5 years of age, the reported incidence of pertussis has risen steadily since 2014, reaching a peak of 2.71 per 100,000 in 2022—the highest level since 1989 (1). During the COVID-19 pandemic, an atypical surge in pertussis cases was observed in the last 2 years, likely due to immunity gaps. According to the China CDC, 32,380 pertussis cases were reported in the first 2 months of 2024, nearly 23 times higher than the number reported during the same period in 2023 (7). It is noteworthy that following the exclusive administration of acellular pertussis vaccines (aPVs) within China’s NIP for children, a significant increase in reported pertussis cases has been observed. This upward trend was particularly evident from 2016 to 2019. Similarly, despite high aPVs coverage in countries such as the United States and Australia, pertussis outbreaks have increased (6, 16, 17). Waining of immunity occurs after aP and wP vaccines as demonstrated by several trials (18, 19). The rise in pertussis outbreaks among both vaccinated and unvaccinated individuals raises concerns about the durability of VE over time.

There are limited studies on the impact of different types of PVs on the incidence of pertussis. To further investigate the reasons for the rebound in the incidence of pertussis, this study aimed to analyze the epidemiological characteristics of pertussis in Linping District and evaluate the VE of PVs in preventing pertussis using a nested case-control research design. Additionally, the study sought to explore potential risk factors for pertussis in a high vaccine coverage setting. The findings aim to provide evidence for the development of immunization policies and strategies for pertussis prevention and control.

## 2 Materials and methods

### 2.1 Data sources

Epidemiological data on pertussis cases and demographic statistics were extracted from the Chinese Disease Control and Prevention Information System (CDCPIS), while vaccination records were obtained from the Zhejiang Province Comprehensive Management Information System for Vaccines and Immunization (ZPCMISVI). These two systems provide complete coverage of notifiable infectious disease reporting and childhood immunization information for all permanent residents in Linping District. The total population denominator data for incidence rate calculation were obtained from the Linping District Bureau of Statistics annual reports.

### 2.2 Case definition

This study included all laboratory-confirmed pertussis cases with a current residential address in Linping District that were reported to the CDCPIS between January 1, 2022 and December 31, 2023.

According to the China’s guidelines for the Diagnosis and Management, and Prevention of pertussis (2024 edition), the following clinical presentations quality patients for inclusion as surveillance cases under hospital passive monitoring: (1) individuals exhibiting paroxysmal spasmodic cough persisting for over 2 weeks without other identifiable causes; (2) infants demonstrating post-tussive symptoms including apnea, asphyxia, cyanosis, or bradycardia. Among these surveillance cases, those showing marked elevation in peripheral white blood cell count with lymphocyte predominance are classified as clinically diagnosed cases. Suspected or clinically diagnosed cases meeting any of the following laboratory criteria are confirmed as pertussis: (1) isolation of Bp from sputum or nasopharyngeal secretions; (2) ≥ 4-fold increase in serum-specific antibody titers between acute and convalescent phases; (3) positive PCR test results. Reporting incidence rates were calculated by dividing the number of the pertussis cases reported among Linping District residents by the total population for 2022 and 2023, then multiplying by 100%.

### 2.3 Vaccines types and immunization schedule

The PV administered in this study comprised DTwP, DTaP, DTaP-Hib, and DTaP-IPV/Hib. Within Linping District, DTwP and DTaP are included in the NIP, following China’s standard vaccination schedule of four 0.5 mL doses administered intramuscularly at 3, 4, 5, and 18 months of age. The DTaP-Hib vaccine follows the same administration protocol as domestic DTaP (1.0 mL intramuscular injection). For DTaP-IPV/Hib, the recommended schedule consists of three 0.5 mL doses at 2, 3, and 4 months of age, followed by a booster dose at 18 months (intramuscular injection). Timely vaccination rates were calculated by dividing the number of people who received the 1st, 2nd, 3rd, and 4th doses by the number of individuals in the cohort at 3 months, 4 months, 5 months, and 18 months of age, respectively, then multiplying by 100%. During 2022–2023, all DTaP vaccines administered in the public sector of Linping District were domestic co-purified acellular pertussis combination vaccines produced by two manufacturers (Wuhan Institute of Biological Products Co., Ltd. and Chengdu Institute of Biological Products Co., Ltd.).

### 2.4 Immunization history

Immunization history were verified using the ZPCMISVI. For subjects whose vaccination records were not found in the system, standardized telephone interviews were conducted by investigators to collect immunization history data. Immunization history was considered valid only for vaccine doses administered before symptom onset and at least 21 days prior; doses given after case reporting or within the 21-day period preceding symptom onset were excluded from the analysis.

### 2.5 Vaccine effectiveness evaluation

Vaccine effectiveness (VE) was determined by calculating the percentage reduction in pertussis reporting incidence rate among vaccinated individuals compared to unvaccinated individuals.

### 2.6 Study object and design

In this study, a nested case-control design was employed within the population-based surveillance cohort to assess the absolute VE of each of four PV types. Case data were obtained from the CDCPIS, where all healthcare institutions in Zhejiang Province report and manage pertussis cases. Given that the vaccination system only contains PV immunization records for individuals under 18 years old and lacks adult vaccination archives, this study limited the participant age range to 0–17 years.

Selective cases: a) Inclusion criteria: pertussis cases with current residential address in Linping District; reported between January 1, 2022 and December 31, 2023; and age < 18 years at reporting. b) Exclusion criteria: children below 3 months of age (minimum immunization age); and duplicate cases (same individual diagnosed at different hospitals); and whose current residence is outside Linping District. After applying these criteria, 138 cases were included. It should be noted that cases aged <3 months and >17 years were included only in the descriptive epidemiological analysis to fully present the disease burden. However, these cases were strictly excluded from the VE estimation in accordance with the eligibility criteria.

Selective controls: a) Inclusion criteria: Children with no reported history of pertussis in the ZPCMISVI source population. b) Exclusion criteria: children below 3 months of age (minimum immunization age); and whose current residence is outside Linping District.

Matching method: A 1:4 individually matched design was adopted. Given that this study focuses on the impact of subtle age differences during infancy, age was strictly matched (within ± 1 month), while gender and township/street were also matched. The matching process was conducted within the ZPCMISVI database: first, all potential controls meeting the above criteria were screened for each case, followed by random sampling to select controls until either four controls were identified or all eligible controls were exhausted, resulting in a total of 552 controls initially screened. Subsequently, the preliminary matching results were reviewed based on the quality control criterion that “the date of birth must precede the case’s onset date.” 26 matched sets were entirely excluded as they failed to meet this condition. Ultimately, 112 valid matched sets were formed, comprising 112 cases and 448 controls included in the final analysis (total sample size *N* = 560).

### 2.7 Statistical analysis

All data processing was performed using Microsoft Excel 2007. We conducted epidemiological analyses of pertussis cases and vaccination data, with VE assessed through a nested case-control study design. Categorical data are presented as *n* (%). Statistical analyses were performed in SPSS 22.0, employing: a) *χ^2^* tests for categorical variable comparisons between groups. b) Conditional logistic regression to calculate odds ratios (OR) with 95% confidence intervals (*95%CI*). c) VE estimation using the formula: VE = (1 − OR) × 100%, where OR approximates relative risk. The significance threshold was set at *α* = 0.05 for all statistical tests.

## 3 Results

### 3.1 Epidemiological characteristics

#### 3.1.1 Distribution of reported pertussis by month

**Figure 1:**
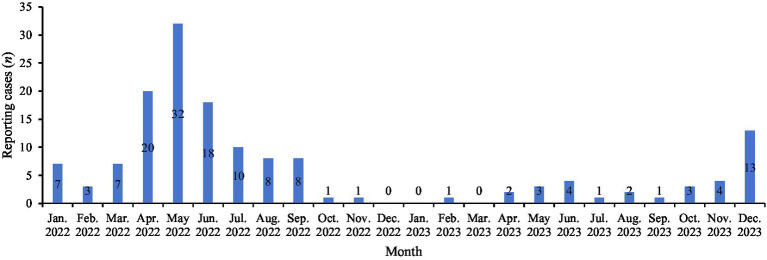
Distribution of reported pertussis by month in Linping District.

The seasonal peak of pertussis in Linping District from 2022 to 2023 was unimodal. Cases were mainly concentrated from April to June 2022 (46.98%, 70/149), while the lowest numbers were recorded in January and March 2023 (0.67%, 1/149). There was a statistically significant difference in the monthly distribution of reported cases (*χ^2^* = 85.13, *p* < 0.001) (Figure 1).

#### 3.1.2 Distribution of reported pertussis by demographic characteristics

From 2022 to 2023, a total of 149 cases of pertussis were reported in Linping District. The reporting incidence rates in 2022 and 2023 was 9.59 per 100,000 population (115 cases) and 2.81 per 100,000 population (34 cases), respectively. The overall reporting incidence rate for pertussis during this period was 6.20 per 100,000 population. Among these cases, the reporting incidence rates for males were 9.35 per 100,000 population (60 cases) in 2022 and 2.78 per 100,000 population (18 cases) in 2023. For females, the reporting incidence rates were 9.87 per 100,000 population (55 cases) in 2022 and 2.84 per 100,000 population (16 cases) in 2023 (Table 1). The hospitalization rates in 2022 and 2023 was 12.17% (14/115) and 26.47% (9/34), respectively, and there were no reported deaths.

**Table 1:** Distribution of reported pertussis by demographic characteristics in Linping District, *n* (per 10^5^).

Age group	2022 year			2023 year			Total		
	Male	Female	Total	Male	Female	Total	Male	Female	Total
<1 yr	13 (257.07)	13 (268.15)	26 (262.49)	6 (130.38)	3 (68.00)	9 (99.84)	19 (193.72)	16 (168.07)	35 (181.17)
1 yr	1 (19.29)	—	1 (9.85)	—	1 (20.56)	1 (10.06)	1 (9.64)	1 (10.28)	2 (9.96)
2 yr	2 (33.20)	2 (35.92)	4 (34.51)	—	1 (20.01)	1 (9.79)	2 (16.60)	3 (27.96)	5 (22.15)
3 yr	2 (31.25)	2 (33.73)	4 (32.44)	1 (16.50)	1 (17.85)	2 (17.15)	3 (23.87)	3 (25.79)	6 (24.79)
4 yr	14 (180.88)	6 (84.02)	20 (134.40)	—	—	—	14 (90.44)	6 (42.01)	20 (67.20)
5 yr	6 (83.93)	6 (91.91)	12 (87.74)	2 (25.68)	2 (27.84)	4 (26.72)	8 (54.80)	8 (59.88)	16 (57.23)
6 yr	6 (104.38)	9 (173.48)	15 (137.16)	3 (41.71)	2 (30.46)	5 (36.34)	9 (73.05)	11 (101.97)	20 (86.75)
7 yr	5 (80.97)	5 (90.17)	10 (85.32)	—	2 (38.32)	2 (18.18)	5 (40.49)	7 (64.25)	12 (51.75)
8 yr	2 (34.34)	4 (77.10)	6 (54.49)	—	1 (17.92)	1 (8.48)	2 (17.17)	5 (47.51)	7 (31.48)
9 yr	5 (79.35)	5 (89.56)	10 (84.15)	1 (17.06)	2 (38.31)	3 (27.08)	6 (48.21)	7 (63.94)	13 (55.61)
10–14 yr.^*^	4 (16.57)	3 (14.42)	7 (15.57)	5 (19.14)	—	5 (10.26)	9 (17.85)	3 (7.21)	12 (12.91)
55–59 yr.^*^	—	—	—	—	1 (2.46)	1 (1.15)	—	1 (1.23)	1 (0.58)
Total	60 (9.35)	55 (9.87)	115 (9.59)	18 (2.78)	16 (2.84)	34 (2.81)	78 (6.07)	71 (6.36)	149 (6.20)

^*^The denominator for the reported incidence rate of pertussis is the total population of the respective age group.

**Figure 2:**
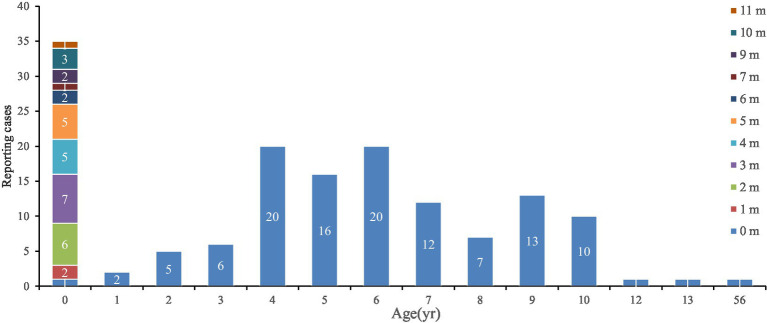
Distribution of reported pertussis by age in Linping District.

Among the 149 reported cases, 78 were males and 71 were females, yielding a male-to-female incidence ratio of 1.10:1. However, there was no significant difference in the incidence between genders (*χ^2^* = 0.66, *p* = 0.417). Only 1 case was an adult, while the remaining 148 cases were children under 18 years old, accounting for 99.33% of the total cases. In terms of age distribution, the 0-year-old group had the highest number of cases, accounting for 23.49% (35/149), followed by the 4-year-old group and the 6-year-old group, each accounting for 13.42% (20/149). There was a statistically significant difference in the distribution of reported cases across different age groups (*χ^2^* = 123.98, *p* < 0.001). Among the reported cases of 0-year-old infants, the highest number of cases was observed in the 3 to 5 months age group, accounting for 48.57% (17/35). This was followed by the 0 to 2 months and the 6 to 11 months age groups, each accounting for 25.71% (9/35). However, there was no statistically significant difference in the distribution of reported cases across different months (*χ^2^* = 14.62, *p* = 0.105) (Figure 2).

### 3.2 Immunization history

#### 3.2.1 Distribution of reported pertussis by vaccination dose

The vaccination history of PV for the 149 reported pertussis cases was verified through the ZPCMISVI. Among them, 22 cases (14.77%) reported no vaccination history prior to the onset of disease, while 127 cases (85.23%) had a vaccination history. Specifically, cases with a history of receiving 1 dose, 2 doses, 3 doses, and 4 doses of the PV accounted for 6.71% (10/149), 0.67% (1/149), 4.70% (7/149), and 73.15% (109/149) of the total cases, respectively. Nine cases (6.04%) of children under 3 months of age had not reached the national starting age for pertussis vaccination and thus had not received the vaccine. Additionally, 23 cases (16.55%) were not vaccinated on schedule among the 139 eligible children (≥3 months). Notably, among children aged 3 to 5 months, 88.24% (15/17) were not vaccinated on time. Among the 112 children who were ≥18 months of age and eligible for vaccination according to the recommended age range, 3 cases (2.68%) failed to complete the full course of 4 doses of the PV (Table 2).

**Table 2:** Distribution of reported pertussis by immunization dose, *n* (%).

Month/age	*N* (%)					
	0 dose	1 dose	2 doses	3 doses	4 doses	Total
0 m	1 (100.00)	—	—	—	—	1 (0.67)
1 m	2 (100.00)	—	—	—	—	2 (1.34)
2 m	6 (100.00)	—	—	—	—	6 (4.03)
3 m	5 (71.43)	2 (28.57)	—	—	—	7 (4.70)
4 m	2 (40.00)	3 (60.00)	—	—	—	5 (3.36)
5 m	2 (40.00)	3 (60.00)	—	—	—	5 (3.36)
6 m	1 (50.00)	—	1 (50.00)	—	—	2 (1.34)
7 m	—	—	—	1 (100.00)	—	1 (0.67)
9 m	—	1 (50.00)	—	1 (50.00)	—	2 (1.34)
10 m	1 (33.33)		—	2 (66.67)	—	3 (2.01)
11 m	—	1 (100.00)	—	—	—	1 (0.67)
12 m	—	—	—	1 (100.00)	—	1 (0.67)
23 m	—	—	—	—	1 (100.00)	1 (0.67)
2 ~ 13 yr	1 (0.90)	—	—	2 (1.80)	108 (97.30)	111 (74.50)
56 yr	1 (100.00)	—	—	—	—	1 (0.67)
Total	22 (14.77)	10 (6.71)	1 (0.67)	7 (4.70)	109 (73.15)	149 (100.00)

#### 3.2.2 Distribution of reported pertussis by post-immunization time and vaccine type

Among the 127 reported cases with a PV immunization history, categorized by the time elapsed since immunization, 69 cases (54.33%) had an elapsed time of 4 ~ 11 years, constituting the largest proportion. Among these 69 cases, 68 cases (98.55%) had completed the full course of 4 doses of vaccination. This was followed by cases with an elapsed time of 1 ~ 3 years since vaccination, accounting for 29.13% of the total. Out of these cases, 36 cases (97.30%) had completed 4 doses of vaccination. Cases with the disease onset within 1 year of vaccination accounted for 16.54%. Among them, 10 cases (47.62%) had only received the first dose of vaccination, while 5 cases (23.81%) had completed either 3 or 4 doses of vaccination.

In terms of the types of PV, four different vaccines were involved. The most commonly used vaccine was the DTaP vaccine, with 88 cases (69.29%) having received this vaccine. Among these 88 cases, 80 cases (90.91%) had completed 4 doses of vaccination. Vaccinations with DTaP-IPV/Hib, DTwP, and DTaP-Hib accounted for 20 cases (15.75%), 18 cases (14.17%), and 1 case (0.79%) of the total, respectively. Among these, the proportions of cases that had completed 4 doses of vaccination were 55.00% (11/20) for DTaP-IPV/Hib, 94.44% (17/18) for DTwP, and 100.00% (1/1) for DTaP-Hib (Table 3).

**Table 3:** Distribution of reported pertussis cases by post-immunization time and vaccine type, *n* (%).

Variable	*N* (%)				
	1 dose	2 doses	3 doses	4 doses	Total
Post-immunization time (year)					
<1	10 (47.62)	1 (4.76)	5 (23.81)	5 (23.81)	21 (16.54)
1–3	—	—	1 (2.70)	36 (97.30)	37 (29.13)
4–11	—	—	1 (1.45)	68 (98.55)	69 (54.33)
Vaccine type					
DTwP	—	—	1 (5.56)	17 (94.44)	18 (14.17)
DTaP	5 (5.68)	—	3 (3.41)	80 (90.91)	88 (69.29)
DTaP-Hib	—	—	—	1 (100.00)	1 (0.79)
DTaP-IPV/Hib	5 (25.00)	1 (5.00)	3 (15.00)	11 (55.00)	20 (15.75)

### 3.3 Vaccine effectiveness evaluation

A total of 112 children were included in a 1:4 matching study, resulting in an overall sample size of 560 (112 cases and 448 controls). The male-to-female ratio was 1.07:1. Age distribution showed that children aged <1 year, 1 ~ 3 years, and 4 ~ 14 years accounted for 10.71, 10.71, and 78.57% of the total sample, respectively (Table 4).

**Table 4:** Baseline characteristics.

Variables	Cases (*n* = 112)	Controls (*n* = 448)	Total (*n* = 560)	%
Sex				
Male	58	232	290	51.79
Female	54	216	270	48.21
Age (year)				
<1	12	48	60	10.71
1 ~ 3	12	48	60	10.71
4 ~ 14	88	352	440	78.57
Address				
Linping subdistrict	7	28	35	6.25
Nanyuan subdistrict	23	92	115	20.54
Donghu subdistrict	11	44	55	9.82
Qiaosi subdistrict	28	112	140	25.00
Xingqiao subdistrict	12	48	60	10.71
Yunhe subdistrict	3	12	15	2.68
Tangqi town	8	32	40	7.14
Chongxian subdistrict	20	80	100	17.86

In the overall population, 98.39% (551/560) had a history of pertussis vaccination, with 93.75% (105/112) in the case group and 99.55% (446/448) in the control group. A total of 87.32% had completed 4 doses of PV, including 86.61% in the case group and 87.50% in the control group. Among the 112 reported cases, the largest proportion (54.82%) had an interval of 4 ~ 11 years since their last vaccination. In the total population, 64.46% had received the DTaP vaccine, with 66.07% in the case group and 64.06% in the control group.

Conditional logistic regression analysis was performed on the vaccination to estimate the VE of the PV in Linping District. The results indicated that the OR of pertussis was 0.071 (*95% CI*: 0.015 ~ 0.344) among those with no immunization history. The protection rate was 92.86% (*95% CI*: 65.62% ~ 98.52%). Specifically, the VE for those with 1–3 doses of basic immunization was 93.84% (*95% CI*: 68.10% ~ 98.81%). The VE within 1 year after PV vaccination was 94.61% (*95% CI*: 71.08% ~ 99.00%). The overall VE of DTwP, DTaP, DTaP-Hib and DTaP-IPV/Hib vaccination was 92.68% (*95% CI*: 60.30% ~ 98.65%), 91.69% (*95% CI*: 59.13% ~ 98.31%), 93.64% (*95% CI*: 7.77% ~ 99.56%) and 94.14% (*95% CI*: 70.45% ~ 98.84%), respectively. Due to the small sample size in the DTaP-Hib group, the estimates were unstable, resulting in extremely wide confidence intervals (Table 5).

**Table 5:** Vaccine effectiveness evaluation of pertussis vaccines.

Variables	Total (*n* = 560)	Case (*n* = 112)	Control (*n* = 448)	*p*-value	OR-value (*95.0% CI*)	VE (%) (*95.0% CI*)
Immunization history, *n* (%)						
No	9 (1.61)	7 (6.25)	2 (0.45)		1.00 (Reference)	
Yes	551 (98.39)	105 (93.75)	446 (99.55)	0.001	0.071 (0.015 ~ 0.344)	92.86 (65.62 ~ 98.52)
Immunization dose, *n* (%)						
0	9 (1.61)	7 (6.25)	2 (0.45)		1.00 (Reference)	
1 ~ 3	62 (11.07)	8 (7.14)	54 (12.05)	0.001	0.062 (0.012 ~ 0.319)	93.84 (68.10 ~ 98.81)
4	489 (87.32)	97 (86.61)	392 (87.50)	0.004	0.086 (0.016 ~ 0.451)	91.42 (54.94 ~ 98.37)
Post-immunization time (year), *n* (%)						
No immunization	9 (1.61)	7 (6.25)	2 (0.45)		1.00 (Reference)	
<1	77 (13.75)	11 (9.82)	66 (14.73)	0.001	0.054 (0.010 ~ 0.289)	94.61 (71.08 ~ 99.00)
1 ~ 3	167 (29.82)	35 (31.25)	132 (29.46)	0.006	0.097 (0.019 ~ 0.504)	90.31 (49.63 ~ 98.14)
4 ~ 11	307 (54.82)	59 (52.68)	248 (55.36)	0.002	0.063 (0.011 ~ 0.356)	93.65 (64.36 ~ 98.87)
Vaccine type, *n* (%)						
No immunization	9 (1.61)	7 (6.25)	2 (0.45)		1.00 (Reference)	
DTwP	84 (15.00)	16 (14.29)	68 (15.18)	0.002	0.073 (0.014 ~ 0.397)	92.68 (60.30 ~ 98.65)
DTaP	361 (64.46)	74 (66.07)	287 (64.06)	0.002	0.083 (0.017 ~ 0.409)	91.69 (59.13 ~ 98.31)
DTaP-Hib	6 (1.07)	1 (0.89)	5 (1.12)	0.043	0.064 (0.004 ~ 0.922)	93.64 (7.77 ~ 99.56)
DTaP-IPV/Hib	100 (17.86)	14 (12.50)	86 (19.20)	0.001	0.059 (0.012 ~ 0.296)	94.14 (70.45 ~ 98.84)

## 4 Discussion

In Linping District, the vast majority of pertussis cases (99.33%) were children aged between 0 and 13 years, with only 1 case being that of an adult. The reporting incidence rates of pertussis in Linping District in 2022 and 2023 was 9.59 per 100,000 population and 2.81 per 100,000 population, respectively. These rates were significantly higher than the national average and those of other provinces during the same period (1, 20, 21). This disparity may be attributed to several factors. Firstly, as Linping District is located in the suburban area of Hangzhou, local residents generally have relatively limited knowledge about pertussis and often lack appropriate preventive measures. Secondly, the shortage of medical resources in the area might result in delays in the diagnosis and treatment of the disease. Consequently, this allows the disease to spread more easily, contributing to an increase in the incidence rate. In addition, this study only confirmed 1 case of adult pertussis, which should be considered as a severe underdiagnosis rather than a truly rare occurrence. This is mainly due to insufficient awareness among medical staff, atypical symptoms in adults leading to diagnostic difficulties, and limited accessibility of local qPCR and serological testing methods, resulting in the true disease burden of adult pertussis in Linping District being almost underestimated. Therefore, it is essential to continuously monitor the epidemic characteristics and evolving trends of pertussis and conduct in-depth exploration of the factors influencing the changes in its incidence.

The COVID-19 pandemic that emerged in late 2019, along with the subsequent non-pharmaceutical interventions implemented for outbreak control, created immunity gaps that significantly transformed the epidemiology of pertussis (22). The monitoring data from Linping District indicated that pertussis cases were mainly concentrated from April to June 2022 (accounting for 46.98%), with the highest peak in May 2022. The third quarter of 2022 ranked second (17.45%), reflecting a trend of more reported cases in summer and autumn. This pattern was largely consistent with the monitoring data from other studies (20, 21). However, the increase in cases of cough caused by other respiratory pathogens in autumn and winter might affect the reporting of atypical pertussis cases.

The most effective means of controlling and preventing pertussis globally is vaccination. Vaccination against pertussis in pregnancy is a useful strategy to protect young infants against the disease through transplacental transfer of anti-pertussis antibodies from the pregnant parent to the fetus (23). In fact, many countries currently recommend that pregnant individuals receive the TdaP vaccine to protect vulnerable infants (24). An Australian study has strongly indicated that improving the timeliness of infant DTaP vaccination, particularly the first dose at 2 months of age, can offer early protection to infants (25). The co-purified DTaP vaccine used in China differs fundamentally in production processes and antigen composition from the component-purified acellular vaccines used in countries such as Australia. In China, the first dose of DTaP vaccination for children is scheduled at 3 months old. In this study, the incidence rate of reported pertussis cases among infants under 1 year old was the highest (181.17 per 105 population), which is consistent with previous domestic and international studies (1, 17, 20, 21). Among these cases, the highest proportion was found in patients aged 3–5 months (48.57%). According to the WHO, a single dose of PV provides approximately a 50% protection rate against severe pertussis in infants. The first dose should be given at 6 weeks of age, with no delay beyond 8 weeks, and three doses of basic immunization should be completed before 6 months of age (26). Results from a study in Shanghai, China, showed that most infants under 1 year old were either unvaccinated against pertussis or not fully vaccinated. This situation can heighten the risk of severe pertussis and complications in infants and young children (27). In this study, 16.55% of children of the appropriate age had reached the first month of immunization but had not received the PV on time. Given that the pertussis vaccination coverage in China is extremely high (>99%), the extremely low pertussis mortality rate (zero deaths) observed in this study may indirectly indicate that the current vaccine is particularly effective in preventing severe disease and death, potentially even more so than in preventing mild illness or infection. Notably, 88.24% of the cases aged 3–5 months had not received the PV in a timely manner. The VE evaluation results demonstrated that the VE for 1 to 3 doses of basic immunization and for completing the full 4-dose series was 93.84% (*95% CI*: 68.10% ~ 98.81%) and 91.42% (95% CI: 54.94% ~ 98.37%), respectively. However, 56 confirmed cases were still identified in the 4–6 years age group in this study, suggesting a trend of waning protection with increasing time since vaccination. Therefore, the current 4-dose DTaP vaccination schedule provides effective protection in early childhood (at least up to preschool age). Future consideration should be given to the feasibility of introducing a booster dose in older children or adolescents. Delayed immunization and non-immunization with the PVs were identified as risk factors for pertussis infection (28). It is of utmost importance to provide vaccine protection to infants as early as possible and to employ various methods to conduct individual follow-up and guidance for unvaccinated children. As of January 1, 2025, the immunization schedule in China shifted from the previous protocol of administering one dose of DTaP Vaccine each at 3 months, 4 months, 5 months, and 18 months of age, along with a dose of the diphtheria and tetanus (DT) vaccine at 6 years old, to a new regimen where one dose of DTaP vaccine is given at 2 months, 4 months, 6 months, 18 months, and 6 years of age. This can lead to a larger proportion of the infant population being protected against pertussis, diphtheria, and tetanus at an earlier stage of life.

In this study, the reported incidence of pertussis among preschool children aged 4 to 6 years increased once again, accounting for 37.58%. Zerbo et al. (29) reported that children aged 19–84 months who had received the aPV had a fivefold higher risk of developing whooping cough 3 years after their last vaccination compared to 1 year after the last vaccination. Currently, in China’s immunization strategy, the last dose of the PV vaccination is administered at 18 months of age. In this study, 97.32% of eligible children aged ≥18 months completed four doses of immunization. However, the VE evaluation results showed no significant difference between those who had received four doses of immunization and the non-immunized group. The main function of the DTaP is to reduce the risk of severe cases and deaths due to pertussis in infants and young children. Nevertheless, evidence suggests that DTaP cannot effectively prevent pertussis infection or block its transmission because the immune persistence lasts for approximately 5 years (26). This study results also indicated that the highest proportion of cases (54.33%) occurred 4 to 11 years after immunization, and 98.55% of these cases had completed four doses of PV vaccination. The VE evaluation revealed that for patients within 1 year after immunization, the VE was 94.61% (*95% CI*: 71.08% ~ 99.00%), and 4 doses of DTaP vaccine can guarantee protection at least until 4–6 years. A case-control study in Canada showed that the VE of PV reached 92% in children aged 2–3 years, and after 1 dose was administered at preschool age, the VE could remain at 90%, which is similar to higher than the results of this study (30). The WHO has also pointed out that in countries using aPV, protection may diminish before the age of 6 (26). Based on the finding of this study that cases continued to accumulate in the 4 ~ 6 years age group, it is recommended to introduce a booster dose in the preschool age group. Furthermore, the new immunization program, with the inclusion of the pertussis component in the 6-year-old dose can boost the waning immunity.

Studies have shown that the VE for preventing the incidence of pertussis and hospitalization in children ≤2 years of age after 4 doses of DTaP was 86.9 and 86.4%, respectively. The overall VE demonstrated an upward trend with an increasing number of administered co-purified DTaP doses, and four doses provided substantial protection (15). A case-cohort study in Switzerland indicated that, among children under 2 years old, the VE of 1 ~ 4 doses of component purified DTaP against pertussis hospitalization was 42, 84, 98, and 100%, respectively (31). These findings suggested that timely and complete vaccination with DTaP can effectively reduce the risk of pertussis-related illness and hospitalization. In high-income countries where preschool booster immunization has been implemented, adolescents and adults have become the main source of pertussis transmission in the community, posing a significant threat to infants (32, 33). However, it should be emphasized that pertussis can be transmitted across all age groups, and controlling its spread also requires attention to older populations and the interruption of asymptomatic transmission chains. Additionally, cocoon immunization, which targets the close contacts of infants and young children, has been shown to reduce the morbidity and mortality associated with pertussis. Moreover, TdaP vaccination during pregnancy significantly alleviates the burden of pertussis in infants and young children (34, 35).

In this study, among the four vaccines, namely DTwP, DTaP, DTaP-Hib, and DTaP-IPV/Hib, DTaP had the highest vaccination coverage rate (64.46%), with 90.91% of children having completed 4 doses of it. The VE of DTaP was 91.69% (*95% CI*: 59.13% ~ 98.31%). The other three vaccines also provided highly effective protection, with the DTaP-IPV/Hib combination vaccine showing the highest VE (94.14%). These findings highlight the significance of exploring the more extensive implementation of PVs in China and strengthening surveillance of pertussis symptoms in adolescents and adults. Such measures may contribute to reducing the incidence of pertussis across the entire population. It is worth noting that whole-cell pertussis (wP) vaccines have been discontinued in China since 2013. Therefore, The small number of wP vaccine recipients in this study were all older individuals vaccinated before 2013. Their age, exposure risk, and immunological background are systematically different from those of the current aP-vaccinated population, making direct comparisons between wP and aP VE estimates methodologically inappropriate. Moreover, due to the small sample sizes of the DTwP group, the DTaP-Hib group, and the DTaP-IPV/Hib group, meaningfully estimate the VE of these three vaccines cannot be performed. DTaP-Hib and DTaP-Hib-IPV vaccines are self-paid vaccines in Linping District. Considering that children from families with better economic and social conditions can access medical services and laboratory tests more easily. In addition, these children may be less exposed to pertussis because they live in areas with lower population density. It should be emphasized that there are extensive differences among countries in immunization schedules, case definitions, sensitivity of surveillance systems, accessibility of diagnostic technologies, clinical awareness of pertussis, and healthcare resource allocation—all of which can contribute to significant heterogeneity in vaccine effectiveness estimates. Therefore, the effectiveness estimates from this study cannot be directly compared with those reported for acellular pertussis vaccines in high-income countries.

### 4.1 Strengths and limitations

Strengths: a) This study links data from two major health information systems, utilizing population-based surveillance data, systematically evaluating the real-world VE of PVs vaccination, with good generalizability of conclusions. b) The use of a nested case-control design minimized selection bias while offering advantages in terms of time and cost efficiency. Limitations: a) Potential underreporting of asymptomatic or mild cases. This can lead to underestimation of the true incidence and the VE of pertussis. This can lead to underestimation of the true incidence and the VE of pertussis. Furthermore, due to limited pathogen diagnostic capacity in primary healthcare facilities, some severe cases or even deaths may not have been included in the surveillance system because timely etiological testing was not performed, leading to an overestimation of vaccine effectiveness. b) Vaccination history primarily relies on information system records, which may be inaccurate or incomplete. c) There may be uncontrolled potential confounding factors (Such as the healthy child effect, wherein healthier children are more likely to receive vaccinations). d) The matching design used in this study may result in a higher vaccination rate in the final case group compared to the original population. This selection bias could lead to an underestimation of the VE. e) This single-center study in Linping District has regional specificities in population, immunization strategies, vaccine types, and healthcare resources, limiting the external validity of its VE estimates. Findings should not be directly extrapolated to regions with different epidemiological or programmatic contexts. Multicenter studies are needed to validate generalizability.

## 5 Conclusion

The incidence of pertussis in Linping District was high, predominantly affecting children, with a distinct spring peak. Delayed or missed vaccination was a key risk factor for infant cases. Under high vaccination coverage, four doses of DTaP provide good protection against pertussis at least through preschool age. However, the occurrence of cases in the 4 ~ 6 year age group indicates waning immunity, necessitating continued surveillance and evaluation of the need for booster doses. The findings suggest that, in addition to improving the timeliness of childhood vaccination, exploring booster doses at school age and implementing life-course immunization strategies are necessary to address the challenge of limited immune persistence.

## Data Availability

The original contributions presented in the study are included in the article/supplementary material, further inquiries can be directed to the corresponding author.
